# The age of onset of pubertal development in healthy Thai girls in Khon Kaen, Thailand

**DOI:** 10.1186/1687-9856-2013-S1-P77

**Published:** 2013-10-03

**Authors:** Nongnapat Jirawutthinan, Ouyporn Panamonta, Sumittra Jirawutthinan, Chatchai Suesirisawat, Manat Panamonta

**Affiliations:** 1 Department of Pediatrics, Faculty of Medicine, Khon Kaen University, Khon Kaen 40002, Thailand; 2 Faculty of Accountancy and Management, Mahasarakham University, Thailand

## 

Onset of puberty has shifted toward a younger age in the 21^st^ century. The useful pubertal assessment in the individual child must be based on recent and reliable reference data from the same population. However, currently representative pubertal data for Thai girls are lacking. Therefore, we determine the current prevalence and mean ages at onset of pubertal characteristics in healthy urban Thai girls in Khon Kaen Province, northeast Thailand.

A cross-sectional study was carried out between January and July 2011. Five hundred and three school girls aged 7 to 16 years were enrolled. All were in good physical health. Stages of breast and pubic hair development were rated on girls by Tanner’s criteria. Assessment was performed by a trained pediatrician. Data on menstruation were collected by the status quo method. The median (range) ages of the onset of thelarche and pubarche were 9.3 (7.8-13.4 ) and 10.8 (8.9-14.5 ) years, with the mean± SD of 10.1± 1.2 and 11.6± 1.2 years, respectively. One hundred and eighteen girls had experienced menstruation. The median (range) age of menarche was 11.6 (10.0-14.0) years and the mean age was 11.6± 0.8 years . The mean ages of pubarche and menarche decreased from the previous study significantly (P < 0.001). These data can be used as the reference of normal pubertal development in Thai girls in Khon Kaen for the purpose of determining precocious or delayed puberty.

